# Low Mannose Binding Lectin, but Not L-Ficolin, Is Associated With Spontaneous Clearance of Hepatitis C Virus After Infection

**DOI:** 10.3389/fimmu.2020.587669

**Published:** 2020-11-11

**Authors:** Jing Zhang, Ning Chen, Zhiyun Chen, Yali Liu, Kai Zheng, Yundong Qiu, Nan Zhang, Junping Zhu, Haibin Yu, Qiushui He

**Affiliations:** ^1^ The Third Unit, Department of Hepatology, Beijing Youan Hospital, Capital Medical University, Beijing, China; ^2^ Department of Medical Microbiology, Capital Medical University, Beijing, China; ^3^ Department of Traditional Chinese Medicine, Gu’an Hospital of Traditional Chinese Medicine, Gu’an, China; ^4^ Department of Medical Microbiology, University of Turku, Turku, Finland

**Keywords:** hepatitis C virus, spontaneous clearance, mannose-binding lectin, L-ficolin, cytokine profile, genotyping, China, cytokine

## Abstract

Some individuals can spontaneously clear the hepatitis C virus (HCV) after infection, whereas others develop a chronic infection. The exact mechanism of this phenomenon is unknown. We aimed to evaluate the association of plasma levels of MBL, L-ficolin, and cytokines with outcome of HCV infections in two groups of patients who cleared HCV spontaneously (CHS), and who developed chronic HCV infections (CHC). Altogether, 86 patients and 183 healthy controls were included. Of 86 patients, 36 had CHS and 50 had CHC. Concentrations of plasma MBL and L-ficolin were measured in patients and controls. Twenty plasma cytokines and adhesion molecules, including GM-CSF, ICAM-1, IFN-γ, IFN-α, IL-1α, IL-1β, IL-10, IL-12p70, IL-13, IL-17A, IL-4, IL-8, IP-10, MCP-1, IL-6, MIP-1α, MIP-1β, sE-Selectin, sP-Selectin, and TNF-α, were determined in all patients and randomly selected 45 controls. The level of MBL was significantly lower in subjects with CHS than in healthy controls (median: 293.10 vs. 482.64 ng/ml, p = 0.008), whereas the level of MBL was significantly higher in patients with CHC than in controls (median: 681.32 vs. 482.64 ng/ml, p = 0.001). No such differences in plasma L-ficolin were observed. Plasma levels of all cytokines and adhesion molecules, except ICAM-1, were significantly higher in patients than in controls. Moreover, patients with CHC had significantly higher levels of IFN-γ, IFN-α, IL-1α, IL-10, IL-13, IL-4, IL-6, and TNF-α than those with CHS. These findings implicate that lower levels of plasma MBL, together with lower levels of above mentioned cytokines may play a part in virus clearance of HCV infection.

## Introduction 

Chronic HCV infection is a global health problem and can progress to liver cirrhosis, hepatocellular carcinoma (HCC), liver failure and even death if untreated (1). In China, the reported incidence of HCV infection was 0.43%, and the incidence in the older age group is higher compared to the younger age group (2). About 10%–46% of infected patients spontaneously clear the virus, while the rest develop a chronic infection (3). However, the mechanisms for spontaneous clearance of HCV are still unclear. Studies showed associations between HCV spontaneous clearing and gene polymorphisms, such as human lymphocyte antigen (HLA) polymorphisms, IFNL3/4 polymorphisms, etc. (4, 5). Virus factors, including HCV genotype, virus heterogeneity, and HCV proteins are also associated with a spontaneous clearance of the virus (3).

Mannose-Binding Lectin (MBL) and L-ficolin are synthesized in the liver and act as soluble pattern recognition molecules (sPRM). MBL and L-ficolin recognize a wide range of microorganisms and activate the complement system through MBL associated serine proteases (MASPs) (6, 7). MBL and L-ficolin are encoded by *MBL2* and *FCN2* genes, respectively. Polymorphisms in *MBL2* and *FCN2* genes have been shown to affect blood protein concentration or oligomer formation (8, 9). A previous study also indicated that serum MBL or L-ficolin activity is associated with the development of HCC in chronic HCV patients (10). And other studies have shown a higher MBL level in chronic HCV patients than in healthy controls (11).

Many studies have shown different cytokine profiles between HCV patients and healthy adults, or between different clinical stages of a HCV infection. However, these results of the cytokine profiles in HCV patients have appeared to be controversial. Baskic et al. (12) suggested lower median values of pro-inflammatory and anti-inflammatory cytokines, including IL-1β, IL-2, IL-17A, etc., in HCV patients than in controls, and the number of participants who had detectable levels of cytokines was remarkably lower in HCV patients. Another study showed elevated concentrations of IL-2, IL-4, IL-10, and IFN-gamma in HCV patients than in controls (13).

Thus, the associations between a spontaneous HCV clearance and the two important sPRMs, MBL, and L-ficolin, and responsive cytokine profiles are still unclear. In this exploratory study, we evaluated whether plasma levels of MBL, L-ficolin, and cytokines are associated with the spontaneous clearance of HCV infection in Chinese adults.

## Materials and Methods

Eighty-six HCV patients and 183 healthy adults were included in this study. All patients lived in Gu’an county, Hebei province and were infected during the donation of their bloods. The patients were enrolled during 2016 and 2018 at the Beijing You’an Hospital. Of the 86 patients, 36 were defined as CHS and 50 as CHC. The definition of CHS was a subject who tested as anti-HCV antibodies positive and RNA of HCV negative without any treatment, and that of CHC was a subject who tested as anti-HCV antibodies positive and RNA of HCV positive without diagnosed HCV-related liver cirrhosis or HCC. In addition, all patients did not have diagnosed systemic diseases such as diabetes, hypertension, heart disease, HIV, cancer, or HBV-related diseases. The control group consisted of 183 healthy adults who attended an annual health examination during 2015–2016. All subjects are Chinese and from different families, and thus considered as unrelated individuals. The study procedures performed were in accordance with the ethical standards of the responsible committee on human experimentation and with the Helsinki declaration of 1975, as revised in 1983. The demographic and clinical information of patients and controls is shown in **Table 1**.

**Table 1:** Demographic and clinical information of patients and controls*.

Subjects (n)	Median (range)	P value 1	P value 2
Controls (n = 183)	48 (22–72)		
CHS (n = 36)	54 (44–72)	<0.001	
CHC (n = 50)	58 (48–76)	<0.001	<0.001
**Male/female**			
Controls	96/87		
CHS	8/28	0.001	
CHC	25/25	0.758	0.009
**HCV RNA (IU/ml)**			
Controls	—		
CHS	<20		
CHC	1,060,000 (2010–18,100,000)		
**ALT(U/L)**			
CHS	16.7 (9.5–52.4)		
CHC	34.4 (6.2–218.9)		0.030
**TBIL(µmol/L)**			
CHS	14.05 (6.5–30.9)		
CHC	14.7 (7.5–39.4)		0.054
**ALB(g/L)**			
CHS	45.75 (40.7–51.7)		
CHC	44.8 (40.5–49.7)		0.576
**HGB(g/L)**			
CHS	136 (7.3–159)		
CHC	142 (98–180)		0.047
**WBC(10^9/^L)**			
CHS	5.66 (2.08–137)		
CHC	5.39 (2.66–10.51)		0.472
**PLT(10^9^/L)**			
CHS	217 (143–401)		
CHC	187 (96–422)		0.018

CHS indicates patients who cleared HCV spontaneously; CHC indicates patients who developed chronic HCV infections and controls indicate those who attended annual health examinations.

*P value 1 indicates the p value obtained from comparison between controls and CHS or CHC groups, respectively; P value 2 indicates the p value obtained from comparison between CHS and CHC groups.

### Determination of Plasma MBL and L-Ficolin

Plasma MBL concentration of all patients and controls was determined as described previously (14) by human MBL ELISA kit (Raybiotech, ELH-MBL, Atlanta, USA), and was performed according to the manufacturer’s instructions. All samples were initially diluted 1:800 and the detection limit was 0.034 ng/ml.

Plasma L-ficolin concentration of all patients and controls was assayed by human ficolin-2 ELISA kit (Raybiotech, ELH-FCN2, Atlanta, USA), and was performed according to the manufacturer’s instructions. All samples were initially diluted 1:400 and the detection limit was 0.123ng/ml.

### Determination of Plasma Cytokines and Adhesion Molecules

For the analysis of 20 cytokines and adhesion molecules, Inflammation 20-Plex Human ProcartaPlex™ Panel (EPX200-12185-901, affymetrix eBioscience, Vienna, Austria) was used. Plasma samples of both HCV patients and controls were thawed and centrifuged at 10,000 x g for 5 min, diluted in 1:2 with diluent (provided by the panel kit), and measured according to the manufacturer’s instructions. Altogether, plasma samples from all patients and randomly selected 45 controls were tested.

### DNA Extraction

Genomic DNAs of the controls and patients were extracted from 200 μl of whole blood, using a DNA purification kit (QIAamp DNA Blood Mini Kit; Qiagen, Valencia, CA, USA), according to the manufacturer’s instructions. The extracted DNAs were stored at −20°C until used.

### Genotyping of *MBL2* and *FCN2* Genes

The six SNPs, rs11003125 (-550, G>C, H/L), rs7096206 (-221, C>G, X/Y), rs7095891 (+4, C>T, P/Q), rs5030737 (codon 52, CGT>TGT, A/D), rs1800450 (codon 54, GGC>GAC, A/B), and rs1800451 (codon 57, GGA>GAA, A/C), of the *MBL2* gene in 82 patients (4 did not have DNA samples available) and 183 controls were detected by PCR-based sequencing using primers 5’-GTAGAGAGGTATTTAGCACTC-3’ and 5’-GCCAGAGAATGAGAGCTGAA-3’ . Of the four SNPs of the *FCN2* gene, rs3124952 (-986, A>G), rs3811140 (-557,A>G), and rs17514136 (-4, A>G) were detected by PCR-based sequencing using primers 5’-AAGTCTTGAGAGGTCTGCC-3’ and 5’-TCAGGGACGAGAAGTTTC-3’, and rs7851696 (+6424, G>T) by PCR-based sequencing using primers 5’-TGCCTCCTGTTCTTCTGTG-3’ and 5’-TCGCACCTTCATCTCTGAC-3’. Eighty-two patients (4 did not have DNA samples available) and all controls were analyzed for the four SNPs of the *FCN2* gene. The reaction volume of each PCR assay was 50 μl, including 5 μl of 10× buffer, 1 μl of each primer (10 μM), 2μl of genomic DNA, 0.5 μl of dNTP mixture, 0.5 μl of high fidelity DNA polymerase and 40 μl of deionized water. The procedure for PCR of *MBL2* SNPs was 5 min at 95°C, followed by 40 cycles (95°C for 30 s, 55°C for 30 s, 72°C for 80 s) and a final extension at 72°C for 10 min. The procedure for PCR of *FCN2* SNPs was 5 min at 95°C, followed by 40 cycles (95°C for 30 s, 58°C for 30 s, 72°C for 80 s) and a final extension at 72°C for 10 min.

### Statistical Analysis

Categorical variables were compared using the Chi-square test or the Fisher’s exact test, as appropriate. Normally distributed continuous variables of the two groups were compared using a student’s T-test. Non-normally distributed continuous variables of the two groups were compared using the Mann-Whitney U-test. All statistical tests were 2-tailed, and a p-value of <0.05 was considered to be significant. All analyses were executed using SPSS software, version 19.0.

## Results

### Demographic Data of Study Subjects

Demographic data of the study subjects are shown in **Table 1**. Subjects in the CHC group had a significantly higher level of alanine aminotransferase (ALT) and hemoglobin (HGB), whereas the level of blood platelet (PLT) was significantly higher in the CHS group than in the CHC group.

### Comparison of Plasma Levels and Genotype Distributions of MBL and L-Ficolin Between Patients and Controls

**Figure 1:**
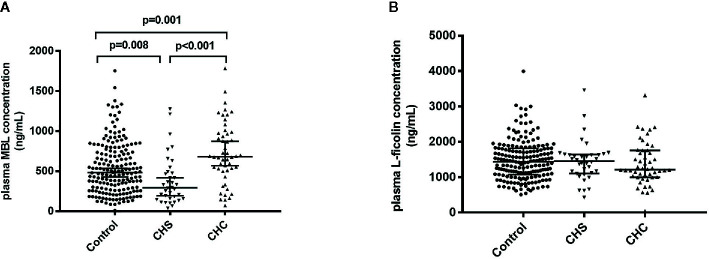
Plasma levels of MBL **(A)** and L-ficolin **(B)** in healthy controls and in patients who spontaneously cleared HCV (CHS) and who developed chronic HCV infections (CHC).

Plasma MBL levels of all patients and healthy adults were determined. The plasma level of MBL was significantly lower in CHS patients compared to controls [median (IQR): 293.10 (158.98 and 489.20) ng/ml vs. 482.64 (169.12 and 745.84) ng/ml, p = 0.008], whereas CHC patients had significantly higher levels of plasma MBL than that of controls [median (IQR): 681.32 (506.88 and 978.34) ng/ml vs. 482.64 (169.12 and 745.84) ng/ml, p = 0.001] (**Figure 1A**). The P value between CHS and CHC patients was less than 0.001. Logistic regression analysis was performed due to the significant difference of the age and gender ratio of study subjects, and the difference in MBL levels among three groups remained significant (p = 0.031, 0.001, and 0.007, respectively). No differences were observed in levels of L-ficolin between controls and CHS patients or CHC patients (**Figure 1B**).

Among the six SNPs of *MBL2* studied, frequency of rs1800450 variant genotype GA was significantly frequent in control group compared with CHC group (24.6% and 10.2% for control group and CHC group, respectively; p = 0.036). Although frequency of rs1800450 variant genotype GA in CHS group was 24.2%, the statistic difference between CHS and CHC groups was not significant. This was most likely due to small sample numbers included in the two groups (**Table S1**). No difference was found in four FCN2 SNPs.

### Association of Genotypes of *MBL2* and *FCN2* With Levels of Plasma Proteins

The *MBL2* and *FCN2* genotypes and their plasma levels were compared in all controls and 82 patients.

*MBL2* genotypes of controls are shown in **Table S2**. The genotype, HYPA/HYPA, was the most common genotype among the controls. Subjects with the genotype A/A had significantly higher MBL levels than those with variant genotype A/B or B/B in control and CHS group (**Figure S1**).

*FCN2* genotypes and plasma L-ficolin levels in control group are shown in **Table 2** and **Figure S2**. Results showed that there are significant associations between the 4 SNPs and plasma L-ficolin levels. For rs3124952 and rs17514136, adults with heterozygous variants had a significantly higher level of L-ficolin, compared to those with the wild-type genotype (**Table 2** and **Figure S2**). However, for rs3811140 and rs7851696, adults with heterozygous or homozygous variants had a significantly lower level of L-ficolin, compared to those with the wild-type genotype (**Table 2** and **Figure S2**). Although the same trend was found in both CHS and CHC groups, the difference did not reach significance.

**Table 2:** Genotypes of FCN2 gene and level of plasma L-ficolin of healthy controls^†^.

SNP	Genotype (n,%) and L-ficolin level median (IQR) (ng/ml)		
rs3124952	GG(149,81.4)	GA(32,17.5)	AA(2,1.1)
	1,336.76(1,007.32–1,740.82)	1,634.18(1,194.62–1,850.11)	2,557.04 and 2,917.92^‡^
rs3811140	AA(126,68.9)	GA(46,25.1)	GG(11,6.0)
	1,560.16(1,214.56–1,889.41)	1,187.22(916.08–1578.48)	809.64(650.16–1,072.56)
rs17514136	AA(155,84.7)	GA(27,14.8)	GG(1,0.5)
	1,361.36(1,054.76–1,747.52)	1,671.00(1,171.48–1,915.52)	255.04^‡^
rs7851696	GG(125,68.3)	GT(47,25.7)	TT(11,6.0)
	1,556.04(1,213.52–1,887.64)	1,189.20(919.16–1,654.44)	809.64(650.16–1,072.56)

^†^N = 183. For plasma L-ficolin, both median and IQR are shown.

^‡^Plasma L-ficolin level of each sample are shown.

### Cytokine and Adhesion Molecule Levels in HCV Patients and Controls

Compared to controls, levels of all cytokines and adhesion molecules, except ICAM-1, were significantly increased in patients (**Table 3**). When levels of cytokines were analyzed in patients with CHS or CHC separately, they were all higher than those of controls, except ICAM-1 (**Tables S3 **and** S4**). Levels of eight cytokines including IFN-γ, IFN-α, IL-1α, IL-10, IL-13, IL-4, IL-6, and TNF-α were significantly higher in patients with CHC than those with CHS (**Table 4**).

**Table 3:** Concentrations (pg/ml) of different plasma cytokines in controls and HCV patients.

Cytokine/Adhesion molecular	control	HCV	P value
GM-CSF^‡^	79.62(63.645-95.54)	113.56(62.69-186.40)	0.003
ICAM-1^‡^	81084.34(55805.845-131001.22)	75147.30(44588.45-114235.32)	0.561
IFN-γ^‡^	12.2(9.07-18.265)	44.37(30.74-60.98)	0.000
IFN-α^‡^	1.39(0.705-2.3)	5.99(4.32-8.23)	0.000
IL-1α^‡^	0.93(0.53-1.6)	3.79(2.21-5.53)	0.000
IL-1β^‡^	13.41(9.88-17.0825)	25.86(17.34-43.61)	0.000
IL-10^‡^	3.74(2.865-5)	11.99(6.76-18.55)	0.000
IL-12p70^‡^	25.8(19.265-30.115)	64.88(44.73-95.22)	0.000
IL-13^‡^	4.94(2.965-6.96)	11.78(8.64-15.16)	0.000
IL-17A^‡^	30.09(22.6-35.1)	49.82(33.81-74.14)	0.000
IL-4^‡^	26.14(19.73-30.755)	60.39(43.72-91.10)	0.000
IL-8^‡^	3.21(2.31-3.67)	8.48(6.03-12.87)	0.011
IP-10^‡^	33.26(22.965-43.6225)	38.52(29.90-52.15)	0.000
MCP-1^‡^	43.78(34.94-51.38)	66.51(50.76-79.16)	0.000
IL-6^†^	46.46(33.16-54.63)	112.73(56.10-198.65)	0.000
MIP-1α^‡^	4.89(3.17-7.75)	11.19(8.43-13.81)	0.000
MIP-1β^‡^	24.72(20.425-33.03)	36.99(26.87-50.50)	0.000
sE-Selectin^‡^	23719.01(20350.24-29589.995)	15272.77(11647.16-20631.42)	0.000
sP-Selectin^‡^	54561.78(38166.04-72633.07)	539481.54(173440.08-1213203.91)	0.000
TNF-α^‡^	17.06(11.975-28.55)	72.8(56.26-103.23)	0.000

^†^Normally distributed continuous variables. Mean concentration (IQR).

^‡^Abnormal distribution continuous variables. Median concentration (IQR).

N = 45 for controls and N = 86 for patients.

**Table 4:** Concentrations (pg/ml) of different cytokines in patients who spontaneously cleared HCV (CHS) and who developed chronic HCV infections (CHC).

Cytokines/Adhesion molecular	CHS	CHC	P value
GM-CSF^†^	115.26(57.65–147.825)	140.66(71.08–191.70)	0.183
ICAM-1^‡^	68,720.44(45,056.51–116,732.54)	82,258.86(43,367.80–114,235.32)	0.889
IFN-γ^‡^	33.92(24.64–46.51)	48.51(37.79–63.39)	0.001
IFN-α^‡^	4.80(3.38–7.46)	6.65(5.31–8.82)	0.003
IL-1α^‡^	2.91(1.79–5.29)	4.12(2.80–5.96)	0.041
IL-1β^‡^	21.17(10.64–38.11)	28.015(18.81–45.12)	0.063
IL-10 ^‡^	7.53(4.06–15.35)	14.21(7.99–20.75)	0.004
IL-12p70^†^	62.70(35.02–85.37)	78.31(49.39–97.72)	0.066
IL-13^‡^	10.17(7.17–12.61)	12.44(9.13–17.00)	0.016
IL-17A^‡^	40.75(30.06–68.36)	57.16(39.68–76.21)	0.083
IL-4^‡^	51.73(35.00–78.26)	66.32(51.49–92.71)	0.009
IL-8^‡^	8.11(5.28–12.39)	8.72(6.49–13.56)	0.207
IP-10^‡^	37.13(26.44–52.86)	39.51(31.04–50.86)	0.457
MCP-1^‡^	71.56(55.67–88.87)	60.8(48.29–77.23)	0.077
IL-6^†^	106.86(38.24–167.14)	146.66(71.65–220.54)	0.043
MIP-1α^‡^	9.89(7.95–13.50)	11.27(9.41–14.07)	0.067
MIP-1β^‡^	33.88(24.39–51.27)	39.29(27.38–50.50)	0.264
sE-Selectin^‡^	16,315.12(13,429.26–24,404.89)	14,289.16(11,076.44–19,454.89)	0.073
sP-Selectin^‡^	317,447.58(114,555.89–1,309,209.36)	544,538.59(231,481.54–1,221,184.48)	0.294
TNF-α^‡^	63.045(46.05–86.24)	96.96(62.46–107.47)	0.000

^†^Normally distributed continuous variables. Mean concentration (IQR).

^‡^Abnormal distribution continuous variables. Median concentration (IQR). N = 36 for CHS group and N = 50 for CHC group.

## Discussion

Our results showed different MBL levels between healthy adults and HCV patients. CHS patients had a lower level, while CHC patients had a higher level, compared to healthy adults. The finding suggested that MBL plays an important role in HCV spontaneous clearance and is in line with earlier studies, in which a higher MBL level in chronic HCV patients compared with healthy controls, as well as a higher MBL level in HCC patients compared with HCV-positive and HCC-negative patients and healthy controls (10, 11). Koutsounaki et al. (15) showed that plasma MBL levels were adversely associated with progression to fibrosis. These studies together with our finding indicate that a higher MBL level may facilitate in progression of HCV disease. However, the exact mechanism of the association MBL has with a spontaneous clearance of HCV is unclear. One possible reason might be that during infection a strong and durable HCV-specific CD4+ and CD8+ T cell response is associated with a spontaneous clearance of the HCV infection (16). It is known that MBL can bind to T cells surface, induce cell arrest in the G0/G1 phase of the cell cycle, and thus suppresses the T cell activation (17).

Researchers showed that L-ficolin inhibits the entry of HCV at an early stage of viral infection, and L-ficolin expression in hepatocytes mediates resistance to HCV infection (18, 19). Study also suggests elevated L-ficolin as a potential biomarker for the development of HCC in chronic HCV infections (10). In this study, we did not find an association between plasma L-ficolin levels and HCV clearance and progression. However, a significant association of FCN2 polymorphisms with production of L-ficolin was observed in healthy adults, which was consistent with previous studies (20, 21).

To assess the cytokine response in HCV patients, we measured 20 different cytokines and adhesion molecules including characteristic ones for Th1, Th2, and Th17 in plasma samples of both patients and controls. The simultaneous elevation of Th1 and Th2 cytokines suggest that both Th1 and Th2 cytokines are associated with pathogenesis of a HCV infection. Many studies showed that Th1 and Th2 cytokines increased in a HCV infection. Fang et al. (22) reported that TH1 cytokine IL-2, and TH2 cytokines IL-4 and IL-10, increased in chronic HCV patients compared to healthy controls, while the TH1 cytokine, IFN-γ was not significantly changed. Sofian et al. (13) showed that levels of IL-2, IL-4, IL-10, and IFN-γ were significantly elevated in HCV patients compared to normal controls. Wright et al. (23) compared 17 cytokines between HCV patients and controls, and the overall cytokine level in HCV patients was significantly higher than in controls. However, the results for the cytokine profile in HCV patients were conflicting. Cribier et al. (24) reported lower level of TH2 cytokines such as IL-4 and IL-6, in HCV patients. Malaguarnera et al. (25) showed lower IFN-γ level in HCV patients, and suggested that a low value of IFN-γ plays a part in HCV chronicity. To determine if cytokine levels of patients with CHS recovered to the normal level as in healthy adults, cytokine levels of these patients and healthy controls were compared. Almost all cytokines measured remained higher in CHS patients than those in the controls, suggesting that the immune status of these patients is still activated. However, compared to CHC patients, CHS patients had decreased levels of certain cytokines, such as IFN-γ, IFN-α, IL-1α, IL-10, IL-13, IL-4, IL-6, and TNF-α, suggesting that these cytokines may play a part in the development of HCV chronicity. Recent studies also showed associations between polymorphisms of IFN-γ, IL-4, and IL-10 polymorphism and HCV clearance. However, the exact effect of these polymorphisms on production of cytokines was unknown (26, 27). It has been shown that the cytokine TNF-α facilitates hepatocyte proliferation and is involved in progress of advanced stages of liver disease (28). Hammad et al. (29) reported IL-6 was associated with an aggravation of the clinical state from HCV infection to cirrhosis, and then to HCC, which is similar to our results. Similarly, Shah et al. (30) reported that greater IL-6 levels are associated with liver fibrosis severity. Altogether, these reports indicate that IL-6 can be a biomarker for aggravation of the HCV infection clinical stage.

According to our result, higher MBL levels and higher cytokine (e.g., IFN-γ, IFN-α, IL-1α, IL-10, IL-13, IL-4, IL-6, and TNF-α) levels may together contribute to a HCV infection being developed into chronic HCV. Thus, the relevance between plasma MBL levels and 20 cytokines were analyzed, and MBL seemed to be positively correlated with IFN-γ, IL-4, and IL-10 in HCV patients (**Figure S3**). Also, we noticed a higher level of IL-10 and sP-selectin in higher MBL producing genotype (A/A) than in lower MBL producing genotype (A/B and B/B) in control group (**Table S5**). According to previous studies, IL-10 acts as a suppressor of T cell proliferation (31). These results indicated that a higher level of IL-10 can promote chronic HCV infection establishment. Although we did not find a significant elevation of IL-10 in CHC patients compared to CHS patients, the IL-10 level was nevertheless higher in the CHC group (median: 18.25 vs. 14.59 pg/ml)

The main limitation to our present study is the small sample size of each patient group. Therefore, the results should be interpreted with caution and we plan to collect more patients’ samples in the future. In addition, cytokines were measured in only a part of control although they were randomly selected.

In summary, our results suggest that lower MBL levels, together with lower levels of certain cytokines such as IFN-γ, IFN-α, IL-1α, IL-10, IL-13, IL-4, IL-6, and TNF-α, may play a part in the virus spontaneously clearing in a HCV infection. Also, higher MBL and these above mentioned cytokines can be aggravation biomarkers for HCV infection outcome.

## Data Availability Statement

The raw data supporting the conclusions of this article will be made available by the authors, without undue reservation.

## Ethics Statement

The studies involving human participants were reviewed and approved by the Beijing Youan Hospital Research Ethics Committee. The patients/participants provided their written informed consent to participate in this study.

## Author Contributions

QH, HY, and JZ conceived, designed, and supervised the study. NC, ZC, YL, KZ, YQ, NZ, and JPZ collected samples and performed the experiments. JZ, NC, HY, and QH analyzed the data and wrote the paper. All authors contributed to the article and approved the submitted version.

## Funding

This work was partly supported by the National 13th five-year Plan Major Infectious Diseases Prevention and Control Science and Technology Major Project (2018ZX10715005-004-006, Beijing Municipal Administration of Hospitals Incubation Program (PX2018058) and Capital's Funds for Health Improvement and Research (2020-1-3011). The funding body had no role in study design, data collection, data analysis or interpretation, or writing of the report. All authors report no conflicts of interest relevant to this article.

## Conflict of Interest

The authors declare that the research was conducted in the absence of any commercial or financial relationships that could be construed as a potential conflict of interest.
