# Supplementary material for: Low Mannose Binding Lectin, but Not L-Ficolin, Is Associated With Spontaneous Clearance of Hepatitis C Virus After Infection
Source: Front Immunol. 2020 Nov 11;11:587669. doi: 10.3389/fimmu.2020.587669 (PMC7686574; doi:10.3389/fimmu.2020.587669)
Supplement: Supplementary file 1 [file DataSheet_1.docx]

**Supplementary materials**

**Table S1. Genotype of rs1800450 in patients and controls**

|  | **CHS, N=33** | | **CHC, N=48** | | **Controls, N=330** | | P value 1 | p value 2 | p value 3 |
| --- | --- | --- | --- | --- | --- | --- | --- | --- | --- |
|  | No. | % | No. | % | No. | % |  |  |  |
| GG(A/A) | 25 | 75.8 | 42 | 85.7 | 136 | 74.3 |  |  |  |
| GA(A/B) | 8 | 24.2 | 5 | 10.2 | 45 | 24.6 | 0.940 | 0.036 | 0.104 |
| AA(B/B) | 0 | 0 | 2 | 4.1 | 2 | 1.1 | 1.000 | 0.247 | 0.231 |

P value 1 indicates the p value obtained from comparison between controls and CHS; P value 2 indicates the p value obtained from comparison between controls and CHC; P value 3 indicates the p value obtained from comparison between CHS and CHC.

**Table S2. Genotype distributions of *M******BL*of healthy controls**

| **Genotype** | **N (%)** |  | **Genotype** | **N (%)** |
| --- | --- | --- | --- | --- |
| HYPA/HYPA | 63 (37.7) |  | LYPA/LYPB | 3 (1.6) |
| HYPA/HYPD | 1 (0.5) |  | LYPA/LYQA | 3 (1.6) |
| HYPA/LXPA | 25 (13.7) |  | LYPB/LXPA | 6 (3.3) |
| HYPA/LYPA | 13 (7.1) |  | LYPB/LYPB | 2 (1.1) |
| HYPA/LYPB | 27 (14.8) |  | LYPB/LYQA | 9 (4.9) |
| HYPA/LYQA | 20 (10.9) |  | LYQA/LXPA | 6 (3.3) |
| LXPA/LXPA | 2 (1.1) |  | LYQA/LYQA | 1 (0.5) |
| LYPA/LXPA | 2 (1.1) |  |  |  |

**Table S3. Levels of cytokine and adhesion moleculesin controls and CHS patients**

| **Cytokine/**  **Adhesion molecule** | **Controls** | **CHS** | **P value** |
| --- | --- | --- | --- |
| GM-CSF ^†^ | 82.10(63.645-95.54) | 115.26(57.65-147.825) | 0.030 |
| ICAM-1^‡^ | 81084.34(55805.845-131001.22) | 68720.44(45056.51-116732.54) | 0.537 |
| IFN-gamma^‡^ | 12.2(9.07-18.265) | 33.92(24.64-46.51) | 0.000 |
| IFN-alpha^‡^ | 1.39(0.705-2.3) | 4.80(3.38-7.46) | 0.000 |
| IL-1alpha^‡^ | 0.93(0.53-1.6) | 2.91(1.79-5.29) | 0.000 |
| IL-1beta^‡^ | 13.41(9.88-17.0825) | 21.17(10.64-38.11) | 0.004 |
| IL-10^‡^ | 3.74(2.865-5) | 7.53(4.06-15.35) | 0.000 |
| IL-12p70^‡^ | 25.8(19.265-30.115) | 62.70(35.02-85.37) | 0.000 |
| IL-13^‡^ | 4.94(2.965-6.96) | 10.17(7.17-12.61) | 0.000 |
| IL-17A^‡^ | 30.09(22.6-35.1) | 40.75(30.06-68.36) | 0.001 |
| IL-4^‡^ | 26.14(19.73-30.755) | 51.73(35.00-78.26) | 0.000 |
| IL-8^†^ | 3.13(2.31-3.67) | 9.04(5.28-12.39) | 0.000 |
| IP-10^†^ | 33.97(22.965-43.6225) | 39.65(26.44-52.86) | 0.090 |
| MCP-1^‡^ | 43.78(34.94-51.38) | 71.56(55.67-88.87) | 0.000 |
| IL-6^†^ | 46.46(33.16-54.63) | 106.86(38.24-167.14) | 0.000 |
| MIP-1alpha^‡^ | 4.89(3.17-7.75) | 9.89(7.95-13.50) | 0.000 |
| MIP-1beta^‡^ | 24.72(20.425-33.03) | 33.88(24.39-51.27) | 0.002 |
| sE-Selectin^‡^ | 23719.01(20350.24-29589.995) | 16315.12(13429.26-24404.89) | 0.002 |
| sP-Selectin^‡^ | 54561.78(38166.04-72633.07) | 317447.58(114555.89-1309209.36) | 0.000 |
| TNF-alpha^‡^ | 17.06(11.975-28.55) | 63.045(46.05-86.24) | 0.000 |

^†^: Normally distributed continuous variables. Mean concentration (IQR); ^‡^: Abnormal distribution continuous variables. Median concentration (IQR).

**Table S4. Levels of cytokine and adhesion molecules in controls and CHC patients**

| **Cytokine**  **Adhesion molecule** | **Controls** | **CHC** | **P value** |
| --- | --- | --- | --- |
| GM-CSF ^†^ | 82.10(63.645-95.54) | 140.66(71.08-191.70) | 0.000 |
| ICAM-1^‡^ | 81084.34(55805.845-131001.22) | 82258.86(43367.80-114235.32) | 0.682 |
| IFN-γ^‡^ | 12.2(9.07-18.265) | 48.51(37.79-63.39) | 0.000 |
| IFN-α^‡^ | 1.39(0.705-2.3) | 6.65(5.31-8.82) | 0.000 |
| IL-1α^‡^ | 0.93(0.53-1.6) | 4.12(2.80-5.96) | 0.000 |
| IL-1β^‡^ | 13.41(9.88-17.0825) | 28.015(18.81-45.12) | 0.000 |
| IL-10^‡^ | 3.74(2.865-5) | 14.21(7.99-20.75) | 0.000 |
| IL-12p70^‡^ | 25.8(19.265-30.115) | 68.35(49.39-97.72) | 0.000 |
| IL-13^‡^ | 4.94(2.965-6.96) | 12.44(9.13-17.00) | 0.000 |
| IL-17A^‡^ | 30.09(22.6-35.1) | 57.16(39.68-76.21) | 0.000 |
| IL-4^‡^ | 26.14(19.73-30.755) | 66.32(51.49-92.71) | 0.000 |
| IL-8‡ | 3.21(2.31-3.67) | 8.72(6.49-13.56) | 0.000 |
| IP-10^‡^ | 33.26(22.965-43.6225) | 39.51(31.04-50.86) | 0.008 |
| MCP-1^‡^ | 43.78(34.94-51.38) | 60.8(48.29-77.23) | 0.000 |
| IL-6^†^ | 46.46(33.16-54.63) | 146.66(71.65-220.54) | 0.000 |
| MIP-1α^‡^ | 4.89(3.17-7.75) | 11.27(9.41-14.07) | 0.000 |
| MIP-1β^‡^ | 24.72(20.425-33.03) | 39.29(27.38-50.50) | 0.000 |
| sE-Selectin^‡^ | 23719.01(20350.24-29589.995) | 14289.16(11076.44-19454.89) | 0.000 |
| sP-Selectin^‡^ | 54561.78(38166.04-72633.07) | 544538.59(231481.54-1221184.48) | 0.000 |
| TNF-α^‡^ | 17.06(11.975-28.55) | 96.96(62.46-107.47) | 0.000 |

^†^: Normally distributed continuous variables. Mean concentration (IQR); ^‡^: Abnormal distribution continuous variables. Median concentration (IQR).

**Table S5. Cytokine level in different MBL genotype in control group**

| **Cytokine/**  **Adhesion molecule** | **A/A** | **A/B or B/B** | **P value** |
| --- | --- | --- | --- |
| GM-CSF^†^ | 85.16(30.26-146.87) | 72.67(50.40-99.25) | 0.125 |
| ICAM-1^‡^ | 77250.24(11433.74-491638.17) | 83734.89(36384.30-153280.22) | 0.428 |
| IFN-gamma^‡^ | 13.60(6.54-25.84) | 10.95(6.54-90.43) | 0.240 |
| IFN-alpha^‡^ | 1.48(0.32-3.75) | 0.80(0.42-11.62) | 0.110 |
| IL-1alpha^‡^ | 1.03(0.08-6.60) | 0.53(0.08-6.45) | 0.098 |
| IL-1beta^‡^ | 13.76(4.87-32.11) | 9.88(5.60-92.73) | 0.091 |
| IL-10^†^ | 4.21(1.98-8.78) | 3.37(1.98-5.00) | **0.036** |
| IL-12p70^‡^ | 26.07(13.19-55.09) | 22.00(13.19-110.71) | 0.135 |
| IL-13^‡^ | 5.06(0.00-15.85) | 4.23(0.00-41.77) | 0.534 |
| IL-17A^‡^ | 31.11(14.27-61.10) | 22.60(16.77-86.37) | 0.066 |
| IL-4^‡^ | 26.14(14.32-51.81) | 22.33(17.06-148.19) | 0.272 |
| IL-8 ^†^ | 3.19(1.29-5.60) | 2.92(1.98-4.46) | 0.483 |
| IP-10^†^ | 33.93(14.78-70.96) | 34.12(16.19-51.96) | 0.968 |
| MCP-1^‡^ | 44.02(24.14-89.27) | 43.72(17.68-158.89) | 0.476 |
| IL-6^‡^ | 50.58(23.34-84.50) | 46.42(28.38-54.63) | 0.145 |
| MIP-1alpha^‡^ | 5.02(0.00-45.53) | 3.64(0.68-14.84) | 0.234 |
| MIP-1beta^†^ | 26.76(13.72-52.46) | 28.43(10.73-63.60) | 0.775 |
| sE-Selectin^‡^ | 23087.86(6135.99-72984.77) | 24623.67(11793.79-41148.08) | 0.279 |
| sP-Selectin^‡^ | 58449.99(28145.22-137729.58) | 30552.00(18311.56-138460.68) | **0.015** |
| TNF-alpha^‡^ | 17.46(5.46-37.46) | 15.45(8.02-137.82) | 0.781 |

^†^: Normally distributed continuous variables. Mean concentration (range); ^‡^: Abnormal distribution continuous variables. Median concentration (range). *p<0.05.

**Figure S1. Plasma MBL level of different MBL genotypes**

A. Plasma MBL level in control group. MBL level (median (IQR)) were 587.68 (417.31-835.38) ng/mL and 253.04 (154.48-353.24) ng/mL for A/A and A/B group, respectively. B. Plasma MBL level in CHS group. MBL level (median (IQR)) were 356.90(195.40-644.25) ng/mL and 175.95(110.75-300.65) ng/mL for A/A and A/B group, respectively. C. Plasma MBL level in CHC group. MBL level (median (IQR)) were 692.24(551.02-978.34) ng/mL and 273.36(154.52-795.80) ng/mL for A/A and A/B group, respectively.

**Figure S2. Plasma L-ficolin level of different FCN2 genotypes**

**Figure S3. Association between MBL and cytokines in HCV patients.**
